# Survival and digestibility of orally-administered immunoglobulin preparations containing IgG through the gastrointestinal tract in humans

**DOI:** 10.1186/s12937-015-0010-7

**Published:** 2015-03-07

**Authors:** Victoria S Jasion, Bruce P Burnett

**Affiliations:** Department of Medical Affairs, Entera Health, 2000 Regency Parkway, Suite 255, Cary, NC 27518 USA

**Keywords:** Serum-derived bovine immunoglobulin, Bovine colostrum, Serum-derived human immunoglobulin, Immunoglobulin digestion, Oral immunoglobulin

## Abstract

Oral immunoglobulin (Ig) preparations are prime examples of medicinal nutrition from natural sources. Plasma products containing Ig have been used for decades in animal feed for intestinal disorders to mitigate the damaging effects of early weaning. These preparations reduce overall mortality and increase feed utilization in various animal species leading to improved growth. Oral administration of Ig preparations from human serum as well as bovine colostrum and serum have been tested and proven to be safe as well as effective in human clinical trials for a variety of enteric microbial infections and other conditions which cause diarrhea. In infants, children, and adults, the amount of intact IgG recovered in stool ranges from trace amounts up to 25% of the original amount ingested. It is generally understood that IgG can only bind to antigens within the GI tract if the Fab structure is intact and has not been completely denatured through acidic pH or digestive proteolytic enzymes. This is a comprehensive review of human studies regarding the survivability of orally-administered Ig preparations, with a focus on IgG. This review also highlights various biochemical studies on IgG which potentially explain which structural elements are responsible for increased stability against digestion.

## Introduction

The biological role of immunoglobulins (Ig) in the protection of the gastrointestinal (GI) tract is well-established, particularly the role of Ig in colostrum and breast milk [1,2]. Hence, colostrum and breast milk are categorized as medicinal nutrition. Due to the need for pasteurization, however, Ig preparations from these sources will have inconsistent amounts of IgG since the amount of IgG denaturation depends upon both the quality of the colostrum and exact method of pasteurization [3]. Efficacy of these formulations in various enteropathies in humans also depends upon the Ig surviving past the stomach into the small and large bowels.

It is generally well-recognized that secretory IgA (sIgA), the primary class of Ig in human colostrum and breast milk as well as being found in the intestinal tract, are stable against enzymatic degradation. Other Igs, such as IgG and IgM, are also less susceptible than typical dietary proteins to digestion, yet this knowledge remains underappreciated despite numerous clinical studies in humans which illustrate recovery of intact and immunologically active IgG from the ileum and feces [4-7]. The purpose of this review is to summarize human clinical studies which assess digestibility of IgG purified from either colostrum or serum in ileal aspirates and stool. This review also summarizes *in vitro* biochemical studies that have assessed structural features of IgG which contribute to their overall stability and discusses for the first time the proposed structural basis for resistance to digestion in the GI tract. With the introduction to the market of the first nutritional therapy in the form of a physician supervised medical food containing high levels of IgG from bovine sera, it is important to understand the pharmacokinetics of these molecules. This understanding is necessary for the usefulness of any Ig-containing formulation as a natural therapeutic for the management of intestinal disorders [8].

## Review

### Immunoglobulin survival through the gastrointestinal tract: *clinical data*

Numerous studies have been performed demonstrating that Ig preparations derived from human sera as well as bovine colostrum and sera survive past the stomach, throughout the GI tract, and are present in fecal matter. These studies are summarized in Table 1. IgG was the predominant Ig in these preparations, but often either IgA and/or IgM was present in smaller amounts.

**Table 1:** **Clinical studies assessing survival of orally-administered immunoglobulin preparations through the digestive tract in humans**

Population (n), health status	Donor species	Preparation	Amount of Ig ingested daily	Material*	Recovery	Recovered immunological activity	Reference
Infant (10), healthy	Bovine	Powder	2 g/kg	IgG (70%), C	13%	Yes (titer correlated with % IgG recovered)	Zinkernagel, et al. (1972) [9]
Infant (6), healthy	Human	Liquid	1 – 8 ml/kg (152 – 1120 mg of IgG)	IgG (~99%), S	4-12%	Yes (titer, 1:2)	Blum, et al. (1981) [10]
Infant (179), low birth weight	Human	Liquid	600 mg	IgA (73%) –IgG (26%), S	1-10 mg/g of dry feces	NR	Eibl et al. (1988) [11]
Infant (164), rotaviral gastroenteritis	Bovine (hyperimmunized)	Liquid	2 g of concentrate/kg	IgG (% NR), M	10%	Yes (titer, 1:48)	Hilpert, et al. (1987) [12]
Children (3), immune deficiency with chronic diarrhea/rotavirus	Human	Liquid	150 mg/kg	IgG (% NR), S	~25%	Yes (titer NR, recovery as intact immune complex, Ig + rotavirus)	Losonsky, et al. (1985) [13]
Children (105), healthy	Bovine (hyperimmunized)	Liquid (one Powder Group)	NR as gram of Ig	Ig (% NR), C	5%	Yes (titer correlated with initial dose)	Pacyna, et al. (2001) [14]
Adult (65), cholera	Bovine (hyperimmunized)	Powder	4 g Ig or 16 g Ig	IgG (~94%), C	10-20%	Yes (titer NR)	McClead, et al. (1988) [17]
Adult (7), healthy	Bovine	Powder	24.4 g Ig	IgG (84%) – IgM (14%), C	~19%^†^	Yes (titer NR)	Roos, et al. (1995) [4]
Adult (6), healthy	Bovine (hyperimmunized)	Powder	2.1 g IgG	IgG (% NR), C	49%^†^	Yes (titer correlated with % IgG recovered)	Warny, et al. (1999) [6]
Adult (6), healthy	Bovine (hyperimmunized)	Powder	14.2 g IgG or 3.4 g IgG	IgG (% NR), C	1.6-32.7%	Yes (titer NR)	Kelly, et al. (1997) [5]
Adult (50), healthy & challenged with *S. flexneri*	Bovine (hyperimmunized)	Liquid	NR as gram of Ig	IgG (% NR), C	ǂ	Yes (titer, ≥ 1:8)	Tacket, et al. (1992) [16]
Adult (72), bone marrow transplant patients	Human	Liquid	50 mg/kg body weight	IgG (% NR), S	1-80 mg/dL of feces	NR	Copelan, et al. (1994) [18]
Adult (12), healthy	Bovine	Powder	NR as gram of Ig	IgG (% NR), S	ǂ	NR	Hanning, et al. (1994) – Unpublished, Data on File.
Adult (4), healthy	Bovine	Powder	0.5 g, 2.5 g or 10 g IgG	IgG (% NR), C	<0.01%	NR	Bogstedt, et al. (1997) [19]
Adult (8), healthy	Bovine	Liquid	7.65 g IgG	IgG, C	<0.1%	No	Lissner, et al. (1998) [20]

^†^Amount collected from the ileum.

ǂ Indicates the presence of immunoglobulin in stool.

*S = serum; C = colostrum; M = milk concentrate.

NR = Not Reported.

#### Recovery in infants

Many of the first published clinical studies on the digestibility of orally-administered IgG were conducted in infant populations. Zinkernagel et al. [9] fed 10 healthy infants at less than three weeks of age a lyophilized bovine colostrum preparation containing 70% IgG administered at 2 g/kg/d. An average 13%-20% of the Ig survived undigested in the stool as demonstrated by an agglutination assay against *E. coli* antigens [9]. In addition, recovered titer correlated with the amount of undigested IgG. In another study, 6 healthy immature, formula-fed infants ingested a 10% human immune serum globulin (HISG), predominantly IgG, in divided doses of 1 to 8 ml/kg/day over 5 consecutive days [10]. The survival of IgG in stool over a 24 hour period ranged between 4-12% of the original IgG ingested. Variability per subject was observed in the survival of active IgG in feces; however, increasing doses were associated with higher amounts of IgG excreted per day. There was no evidence of systemic absorption or adverse events.

In a larger randomized, controlled clinical trial, low-birth weight infants unable to breast feed were administered 600 mg daily of serum-derived human IgA (73%) and IgG (26%). The test group (n = 91) ingested the serum-derived IgA-IgG mixed into either infant formula or infant formula combined with pooled, pasteurized human milk. The control group (n = 88) ingested the same formula/milk preparation, less the serum-derived IgA-IgG [11]. The infants receiving oral IgA-IgG had fewer cases of necrotizing colitis (0 cases) compared to the controls (6 cases) and had “substantial amounts” of intact IgA and IgG recovered in stool compared to the controls. As in the previous study, there was no evidence of systemic absorption.

Bovine milk Ig concentrate purified from hyperimmunized cows against four human rotavirus serotypes has also been studied in a group (n = 164) of low birth weight infants [12]. The infants were dosed at 2 g Ig concentrate/kg/day for five days. Of the infants receiving Ig, stool samples from 47% had detectable bovine IgG and 43% maintained rotavirus-neutralization activity against bovine rotavirus V1005, human rotavirus Wa (serotype 1) and simian rotavirus SA-11 in cell culture. Furthermore, the infants with high amounts of neutralizing activity still present in feces demonstrated clinical benefit [12].

#### Recovery in children

One small (N = 3) and another larger study (N = 105) have been performed to assess recovery of active IgG against rotavirus from feces of children [13,14]. The smaller study was comprised of three pediatric patients ages 16 months and 4 yr, both with severe combined immunodeficiency disease, and 18-yr-old with common variable immunodeficiency disease [13]. All three had a history of intermittent positive excretion of rotavirus serotype 1 with chronic diarrhea, decreased weight gain and fat malabsorption. The children ingested a single dose of 150 mg/kg human sera Ig (IgG at 50 mg/ml) labeled with ^125^I. Approximately 50% of the recovered radioactivity was excreted in the stools over a 3 d period. Half of the excreted radioactively labeled IgG, or 25% of the originally ingested IgG, retained immunological activity, as determined by the recovery of ^125^I-labeled Ig bound to rotavirus [13].

In the second study, children drank 100 ml of whole cow’s milk supplemented with hyperimmunized bovine colostrum against rotavirus, 3 times daily for a period of 6 days [14]. There were five groups based on the rotavirus-antibody titer of the Ig formulation: control (no rotavirus antibody titer), 1:2,500, 1:5,100, 1:8,000, and 1:8,200. After pooling results from the four experimental groups, approximately 5% of IgG was recovered while the level of antibody activity varied considerably. Approximately 88% of the experimental patients had detectable neutralization signals which correlated (r = 0.81) with percent reduction of rotavirus from stools and the titer of ingested milk/colostrum.

#### Recovery in adults

Two studies in adults measured the ileal recovery of orally-administered Igs [4,6]. Healthy, fasted subjects (n = 7) drank 400 ml of a ^15^N-labeled bovine colostrum-derived Ig fraction, containing approximately ~5.2% IgG, 0.86% IgM, and 0.1% IgA (~2 g IgG, 0.34 g IgM, and 0.04 g IgA) and ileal effluents were collected every 20 min for 8 hrs [4]. Approximately 19% of IgG and IgM was still immunologically active from ileal effluent, although the titer was not reported. The authors also reported that 59% of IgG collected from 2 subjects in the jejunum remained active. Further purification of the ileal chyme samples using protein chromatography demonstrated that the fractions with the most immunological activity had a molecular weight of ~100 kDa (kilo-Dalton) which correlates well with *in vitro* pepsin and trypsin digestion experiments of the intact IgG (~160 kDa) into Fab_1,2_ dimers (~100 kDa) and monomers (~50 kDa) [15]. Thus, in addition to demonstrating IgG survivability through the gastric environment into the ileum, these data establish that *in vivo* IgGs are digested in a step-wise manner into active, intact Fab fragment that still bind target antigen (s).

In the second ileal recovery study, 6 healthy volunteers with an end ileostomy ingested 5 g of bovine-Ig concentrate from hyperimmune bovine colostrum against *C. difficile* which contained 2.1 g of IgG alone, with an antacid, during treatment with omeprazole (a proton-pump inhibitor which decreases the acidity of the stomach), or within enteric-coated capsules in four separate experiments [6]. The difference in IgG recovered was not statistically significant: alone (49%), antacid (30%) and omeprazole (50%) (p = 0.13). Counterintuitively, enteric encapsulation resulted in statistically less IgG reaching the ileum (4%) during the time course, compared with both the alone and omeprazole group. Some capsules were recovered intact or partially digested, suggesting that enteric encapsulation inhibited release of IgG in the small intestine. Percent recovery of IgG correlated with neutralizing activity of toxin A. These two studies illustrate that a high percentage of orally-administered IgG (between ~19% and ~50%) can be recovered intact and active from the distal ileum in adult humans before entering the large intestine.

Fecal recovery of orally-administered Igs has also been demonstrated in healthy adults. In one study, healthy adult volunteers (n = 6) took a single oral dose of bovine Ig concentrate (BIC, 45 g with 14.2 g of IgG) from the colostrum of hyperimmunized cows against *C. difficile* [5]. Each subject had a 14-day wash-out period between crossing-over into one of six testing groups: fasting (45 g BIC), fed (45 g BIC), fed (8 g BIC), co-administered with antacid (45 g BIC), with omeprazole (45 g BIC), and enteric-coated capsules (8 g BIC) designed to release the product at pH > 6 in the intestinal tract. Total bovine IgG and specific anti-*C. difficile* IgG activity were measured in feces. The bovine IgG concentration of the fasting group compared to the ingested dose of 45 g BIC after 72 hr in feces was 3.8%, for fed 1.6%, and for antacid 2.7%. Omeprazole increased fecal bovine IgG levels to 8.8%, although not statistically significant over the other groups. In this study, the stool samples from enteric-coated capsules had 32.7% of the IgG in the original dose, 8 g BIC. For the fed group ingesting 8 g BIC, the recovery was 0.6% the original dose. *C. difficile* neutralizing activity correlated with the percentage of IgG recovered. This study showed that enteric encapsulation of oral Ig correlated with higher amounts of IgG recovered in stool and therefore delivered more intact IgG to the colon in healthy adults. Further studies are required to determine whether enteric encapsulation is necessary to protect orally delivered Ig for efficacy in specific intestinal disorders.

Tacket et al. [16] administered two specific preparations of bovine colostrum against *Shigella flexneri* 2a strain 5427 T liposaccharide, anti-LPS IgG which varied by titer of anti-LPS (1:2,560 and 1:40,960, respectively) and addition of chocolate dairy protein powder (1:40,960 titer only) to healthy adults (n = 50). The healthy adults were challenged with 10^3^*c.f.u.* of *S. flexneri* 2a strain 2457 T after three days of ingesting the Ig concentrates above. Bovine IgG was detected in feces at 91% (1:2,560) and 60% (1:40,960) of the original amounts. In both groups, the recovered titer of bovine anti-LPS was ≥ 1:8. This study illustrated a dose–response effect since the group with the higher titer was better protected from *S. flexneri* challenge. In another report of two trials, 2 g of bovine Ig concentrate from the colostrum of cows hyperimmunized against cholera toxin was administered to patients with active cholera diarrhea under different protocols: two doses (4 g bovine Ig concentrate, n = 45) or a single dose for every two hours for a total of eight doses (16 g of Ig concentrate, n = 20). From the 65 patients, a total of 35 individual stool samples were analyzed for bovine IgG and IgA. Low levels of active Ig, either as whole IgG or Fab fragments, was found in stool for roughly 60% of the patients ingesting bovine Ig. An average of about 10-20% cholera toxin neutralizing activity was still present in the recovered stools, although the titer was not reported [17]. In another randomized, controlled trial, patients undergoing bone marrow transplantation (N = 72) who received either 50 mg/kg human gammaglobulin or placebo daily in four divided doses for 28 days after the procedure (500 mg weekly), there was 1 to 80 mg/dL of IgG present in stool. Antibody reactivity was not reported [18].

Other trials reporting only trace amounts of IgG in feces have also been performed. In an unpublished study of 12 healthy adults, when 10 g of serum-derived bovine immunoglobulin/protein isolate (SBI) was given on two consecutive days followed by 2.5 g for 14 days, there were only low levels of detectable IgG in feces [Hanning, R. and M. Drew, *Bovine Immunoglobulin Feeding Trial.* Data on File, 1994]. There was no detectable bovine Ig in the serum of any subjects again supporting the notion of no intact systemic circulation of bovine-derived Ig. In another study, when 0.5, 2.5, or 10 g of bovine IgG was administered to 4 healthy adults, only 0.01% of the ingested IgG was detected in feces. Neutralizing activity of this recovered fraction was not assessed [19]. Similarly, when a 15 g colostrum preparation from non-immunized cows with an IgG concentration of 51% was given to healthy volunteers (n = 8), there was no evidence of systemic absorption of bovine IgG (blood or urine) and in 3 of the 8 patients there were only trace amounts of bovine IgG detected in feces, but without antibody reactivity to *Yersinia enterocolitica* and *Campylobacter jejuni* antigens [20]. These data suggest that the study population may be an important determinant in recovery Ig and neutralizing activity. In healthy individuals with normal transit times, there appears to be less recovery of orally administered Ig compared to patients with accelerated intestinal transit due to infection or disease.

Recovered IgG values varied from study to study. This is not surprising since each study had different patient populations and various immunoglobulin preparations. Yet, these data summarized above strongly suggest that IgG are more resistant to complete digestion throughout the human GI tract than other dietary proteins since only three of the fifteen reports illustrate trace amounts of IgG recovered. It should be mentioned that with respect to nutrition literature, the term “digestibility” is most recently defined as the net absorption of an amino acid [21]. In this review, we are using the term “digestibility” to discuss the integrity of the quaternary and tertiary structure of IgG as it passes through the GI tract in humans, as it is generally understood that IgG can only bind to antigens within the GI tract if the antigen binding domain, the Fab, is intact. This would mean the Fab domain must resist complete denaturation through acidic pH and complete digestion by proteolytic enzymes. Most of the human studies summarized above for IgG assessed crude protein digestibility because they reported a percentage of intact IgG recovered, either from the ileum or from feces. As aforementioned, the many nutrition experiments which assess the digestibility of other dietary proteins are reported as percentages as a function of the net absorption of an amino acid. Therefore, it is difficult to compare the digestibility of IgG as reported in this review (crude protein) to the digestibility of other dietary proteins reported in literature (ileal digestibility as absorbed amino acids). In order to provide some sort of comparable measure, the true ileal digestibility - as defined as absorbed amino acids - of other dietary proteins in humans are: 95% for milk proteins, 94.1% for casein, 90% for pea protein, 91.5% for wheat protein [22]. There is one study reviewed in this manuscript which could conservatively be compared to the above numbers. Roos et al. [4], reported a prececal nitrogen absorption from the Ig preparation to be at 79%, below the absorption percentages mentioned above for other dietary proteins. In addition, recovered active fragments corresponding to ~100 kDa or two Fab domains were purified from the recovered chyme. This study therefore directly corroborates the step-wise process by which IgG are digested by enzymes *in vitro* also occurs in humans *in vivo* and that Ig preparations are digested more slowly, corresponding to less amino acid absorption *via* nitrogen monitoring. There are biochemical *in vitro* experiments which have assessed if various structural features of IgG contribute to overall stability. It is useful to discuss such experiments insofar that the majority of clinical studies reviewed in this manuscript illustrate a percentage of IgG and the Fab domains retained neutralizing activity albeit at a reduced level and more importantly, that a percentage of IgG and Fab domains retain their structural integrity, remaining intact.

### Immunoglobulin structure

**Figure 1:**
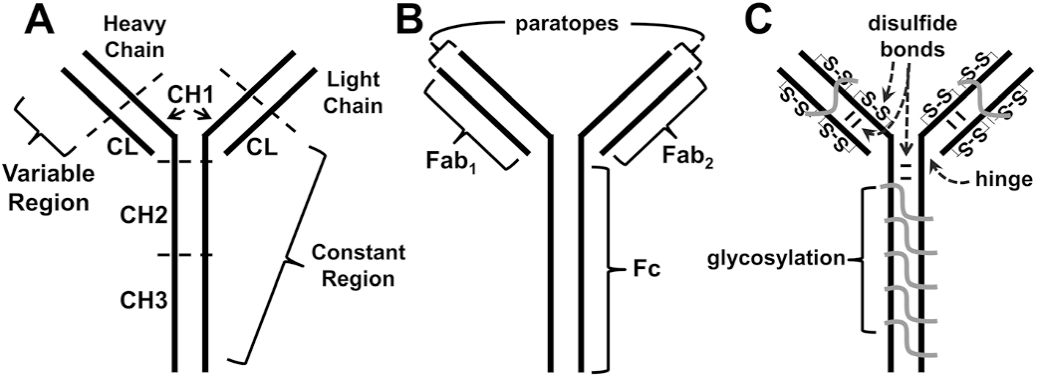
**Simplified two-dimensional schematics of immunoglobulin G (IgG). A**. Schematic showing the variable and constant regions of the IgG molecule for the heavy (CHx) and light chains (CL); **B**. Schematic of the antigen binding fragments (Fab_1,2_) and the fragment crystallizable region (Fc) of the IgG molecule; **C**. Schematic portraying intra- and interchain disulfide bonds as well as glycosylation of the Fab, Fc regions, and paratope antigen binding regions of the IgG molecule.

The base structure of Ig can be represented as a two-dimensional “Y” (Figure 1). The IgG molecule can be divided into two independent active domains: the variable antigen binding domain or fragment and the constant region. The antigen binding fragment, or Fab, is responsible for interacting with the target antigen or epitope *via* binding by the paratope or complementary determining region (CDR) (Figure 1B). IgGs are able to bind to various antigens with high specificity due to the variability of amino acids in the CDR, since the general folded Y-shape (tertiary structure) of Igs is conserved. The fragment crystallizable region (Fc) is an effector to initiate an immune response, and has no antigen binding ability (Figure 1B).

There are two post-translational structural features of Ig molecules which contribute to the overall stability of the molecule: intra- and interchain covalent disulfide bonds between cysteine residues and glycosylation (Figure 1C). Disulfides bonds are hallmark features of Ig stability and are also known to increase the stability of other proteins [23-28]. Other studies link IgG glycosylation, a variable feature for the Fab domain, but a conserved feature on the Fc domain, with stabilizing effects [29-32]. The region most susceptible to enzymatic degradation is the hinge region, which connects the Fab and Fc domains. The hinge region is the most flexible region of the IgG since it is the only single polypeptide region of IgG. Studies on the susceptibility to degradation of IgG to acidic conditions (the stomach) and digestion by enzymes (stomach and small intestine) are reviewed below.

#### Temperature and pH – in vitro

The protein melting temperature (T_M_) is the point at which a protein unfolds, or denatures, and lacks the structure necessary for activity. T_M_ is a biochemical measurement to probe protein structure in different situations, such as pH. Thermal stability of an IgG correlates with pH: lower pH values, 2.8-3.4, corresponded to lower average global T_M_ values, 43.7°C-53.6°C, and higher pH, 5.0-7.5, corresponded to higher average global T_M_ values, 67.5°C-68°C [33]. Even in the acidic pH range of 2.8-3.4, the T_M_ value does not fall below body temperature, 37°C, accounting for Ig structural integrity in the digestive tract. The addition of sugars, trehalose [34] and sucrose [35] to experimental buffers increased T_M_ values for all IgGs with pH values ranging from 4.0-8.0 suggesting that other environmental factors within the GI tract, like dietary molecules, could increase Ig stability. Salt content does not appear to affect IgG stability [35]. There have been isolated reports where the CH2 domain, the most thermally stable domain of the IgG, exhibits increased thermal stability after exposure to pH of 2.0 [36]. This increased stability is correlated with alternate conformations that various IgGs exhibit at this low pH [37-39]*.* Some IgG purification procedures, either from recombinant expression methods used to produce commercially-available biologic drugs or from naturally occurring sources, such as serum, require acidic or basic exposure steps [40,41]. IgG molecules purified utilizing pH shifts do not lose their capacity to bind antigens. In fact, certain intravenous immunoglobulin (IVIg) preparations purified with a low-pH step have enhanced *in vitro* and *in vivo* binding to antigens that correlates with better performance in mouse models of sepsis [41-43]*.* These studies underscore that IgG remain stable in acidic conditions at body temperature. It is also possible that transient exposure to acidic conditions in the stomach may increase stability for survival down the GI tract and to proteolytic enzymes.

#### Resistance to proteolytic digestion

Characterization of IgG structure using *in vitro* digestion with proteolytic enzymes dates to the 1960s [44-47]. It is well-established that enzymatic digestion of IgG at the hinge region with papain produces two active domains, Fab_1,2_ and Fc (Figure 1BC). Furthermore, modulating reaction time, pH and temperature during enzymatic digestion will result in a variety of active domain fragments [48]. The primary digestive enzymes in humans for proteins are pepsin, in the stomach, followed by trypsin and chymotrypsin, in the small intestine [49] which digest IgG to Fab dimers (~100 kDa) and monomer Fab fragments (~50 kDa) [15], but this enzymatic susceptibility may vary by IgG subtype and species. Bovine IgG_1_, for example, is more readily proteolyzed by pepsin than bovine IgG_2_ [50]*.* Bovine IgG is more stable to proteolytic digestion compared to rabbit or human IgG [29]*.* Even after proteolytic digestion of IgG, Fab dimers and monomer fragments retain binding and antigen-neutralizing activity as long as they are not denatured. Proteolytic characterization of bovine derived IgG_1_*in vitro* demonstrates intact and reactive Fab domains [29,50-53]. This correlates with the human *in vivo* study by Roos et al. [4] demonstrating that immunologically active bovine Fabs were recovered in ileal aspirates.

## Conclusion

Twelve of the 15 human studies in infants, children and adults that clearly demonstrate that orally-administered Ig (particularly IgGs), from human and bovine serum as well as from bovine colostrum and milk, survive gastric exposure and resist proteolytic digestion in the stomach and intestinal tract (Table 1) [4-6,9-14,16-18]. The stability of IgG is based on the structural properties of the molecules with contributions from intra- and interchain disulfide bonds, post-transcriptionally added sugars, and the three dimensional folded domains. Even when partially digested by proteolytic enzymes, Fab fragments retain not only binding but neutralizing activity through the digestive tract. In addition, there is no evidence of intact absorption of the protein, making oral IgG administration a safe, potentially effective therapy in a number of GI conditions and diseases. These physical properties and the ability of digested IgG fragments, Fab monomers and Fab_1,2_ dimers to retain active binding activity make them attractive natural therapeutic options for GI conditions [54] and mitigation of damage caused by bacterial enterotoxins, endotoxins and secreted exotoxins [55].

Studies in animals have illustrated that ingestion of serum-derived Ig preparations, containing primarily IgG, increase anti-inflammatory cytokines and decrease pro-inflammatory cytokines in mucosal jejunum [56,57]. Additionally, administration of serum-derived IgG has been shown to prevent increased intestinal permeability induced by enterotoxin challenge [58]. Improved nutrient utilization in animal models and human clinical studies after ingestion of serum-derived immunoglobulin preparations have also been observed [59,60]. More recent studies have also shown efficacy of oral serum-derived bovine immunoglobulin preparations, primarily containing IgG, in such conditions as irritable bowel syndrome with diarrhea (IBS-D) and HIV-associated enteropathy [59,61]. Other patients with chronic conditions, such as inflammatory bowel disease (IBD) or common variable immunodeficiency (CVID) might also benefit from orally administered Ig preparations, containing IgG, due to antigen-neutralizing activity as well anti-inflammatory properties of these preparations. In short, there is sufficient evidence illustrating that IgGs are less susceptible to digestion throughout the GI tract and therefore may provide for a distinctive nutritional requirement unique to patients with intestinal disorders and diseases which other dietary proteins cannot provide.

## Abbreviations

Ig: Immunoglobulin(s)
IgG: Immunoglobulin G
GI: Gastrointestinal
sIgA: Secretory IgA
HISG: Human immune serum globulin
kDa: Kilo-Dalton
BIC: Bovine Ig concentrate
*S. flexneri*: *Shigella flexneri*
SBI: Serum-derived bovine immunoglobulin/protein isolate
Fab: Antigen binding fragment
CDR: Complementary determining region
Fc: Fragment crystallizable region
T_M_: Protein melting temperature
IBS-D: Irritable bowel syndrome, with diarrhea
IBD: Inflammatory bowel disease
CVID: Common variable immunodeficiency

## References

[1] Weiner C, Pan Q, Hurtig M, Boren T, Bostwick E, Hammarstrom L (1999). Passive immunity against human pathogens using bovine antibodies. Clin Exp Immunol.

[2] Hurley WL, Theil PK (2011). Perspectives on immunoglobulins in colostrum and milk. Nutrients.

[3] Godden SM, Smith S, Feirtag JM, Green LR, Wells SJ, Fetrow JP (2003). Effect of on-farm commercial batch pasteurization of colostrum on colostrum and serum immunoglobulin concentrations in dairy calves. J Dairy Sci.

[4] Roos N, Mahe S, Benamouzig R, Sick H, Rautureau J, Tome D (1995). 15 N-labeled immunoglobulins from bovine colostrum are partially resistant to digestion in human intestine. J Nutr.

[5] Kelly CP, Chetham S, Keates S, Bostwick EF, Roush AM, Castagliuolo I (1997). Survival of anti-Clostridium difficile bovine immunoglobulin concentrate in the human gastrointestinal tract. Antimicrob Agents Chemother.

[6] Warny M, Fatimi A, Bostwick EF, Laine DC, Lebel F, LaMont JT (1999). Bovine immunoglobulin concentrate-clostridium difficile retains C difficile toxin neutralising activity after passage through the human stomach and small intestine. Gut.

[7] Bhol KC, Tracey DE, Lemos BR, Lyng GD, Erlich EC, Keane DM (2013). AVX-470: a novel oral anti-TNF antibody with therapeutic potential in inflammatory bowel disease. Inflamm Bowel Dis.

[8] Petschow BW, Burnett BP, Shaw AL, Weaver EM, Klein GL (2015). Dietary Requirement for Serum-Derived Bovine Immunoglobulins in the Clinical Management of Patients with Enteropathy. Dig Dis Sci.

[9] Zinkernagel RM, Hilpert H, Gerber H (1972). The digestion of colostral bovine immunoglobulins in infants. Experientia.

[10] Blum PM, Phelps DL, Ank BJ, Krantman HJ, Stiehm ER (1981). Survival of oral human immune serum globulin in the gastrointestinal tract of low birth weight infants. Pediatr Res.

[11] Eibl MM, Wolf HM, Furnkranz H, Rosenkranz A (1988). Prevention of necrotizing enterocolitis in low-birth-weight infants by IgA-IgG feeding. N Engl J Med.

[12] Hilpert H, Brussow H, Mietens C, Sidoti J, Lerner L, Werchau H (1987). Use of bovine milk concentrate containing antibody to rotavirus to treat rotavirus gastroenteritis in infants. J Infect Dis.

[13] Losonsky GA, Johnson JP, Winkelstein JA, Yolken RH (1985). Oral administration of human serum immunoglobulin in immunodeficient patients with viral gastroenteritis. A pharmacokinetic and functional analysis. J Clin Invest.

[14] Pacyna J, Siwek K, Terry SJ, Roberton ES, Johnson RB, Davidson GP (2001). Survival of rotavirus antibody activity derived from bovine colostrum after passage through the human gastrointestinal tract. J Pediatr Gastroenterol Nutr.

[15] Turner MW, Bennich HH, Natvig JB (1970). Pepsin digestion of human G-myeloma proteins of different subclasses. II. Immunochemical investigations of the products of peptic digestion. Clin Exp Immunol.

[16] Tacket CO, Binion SB, Bostwick E, Losonsky G, Roy MJ, Edelman R (1992). Efficacy of bovine milk immunoglobulin concentrate in preventing illness after Shigella flexneri challenge. Am J Trop Med Hyg.

[17] McClead RE, Butler T, Rabbani GH (1988). Orally administered bovine colostral anti-cholera toxin antibodies: results of two clinical trials. Am J Med.

[18] Copelan EA, Bechtel TP, Klein JP, Klein JL, Tutschka P, Kapoor N (1994). Controlled trial of orally administered immunoglobulin following bone marrow transplantation. Bone Marrow Transplant.

[19] Bogstedt AK, Hammarstrom L, Robertson AK (1997). Survival of immunoglobulins from different species through the gastrointestinal tract in healthy adult volunteers: implications for human therapy. Antimicrob Agents Chemother.

[20] Lissner R, Thurmann PA, Merz G, Karch H (1998). Antibody reactivity and fecal recovery of bovine immunoglobulins following oral administration of a colostrum concentrate from cows (Lactobin) to healthy volunteers. Int J Clin Pharmacol Ther.

[21] Moughan P: Dietary protein quality evaluation in human nutrition: Report of an FAO Expert Consultation. http://www.fao.org/ag/humannutrition/nutrition/63158/en/: FAO; Accessed 5 January 2015. 26369006

[22] Moughan P, Gilani S, Rutherfurd S, Tome D: The assessment of amino acid digestibility in foods for humans and including a collation of published ileal amino acid digestibility for human foods. http://www.fao.org/ag/humannutrition/nutrition/63158/en/: FAO; Accessed 5 January 2015.

[23] Goto Y, Hamaguchi K (1979). The role of the intrachain disulfide bond in the conformation and stability of the constant fragment of the immunoglobulin light chain. J Biochem.

[24] Goto Y, Hamaguchi K (1982). Unfolding and refolding of the reduced constant fragment of the immunoglobulin light chain. Kinetic role of the intrachain disulfide bond. J Mol Biol.

[25] Ashikari Y, Arata Y, Hamaguchi K (1985). pH-induced unfolding of the constant fragment of the immunoglobulin light chain: effect of reduction of the intrachain disulfide bond. J Biochem.

[26] Thies MJ, Talamo F, Mayer M, Bell S, Ruoppolo M, Marino G (2002). Folding and oxidation of the antibody domain C(H) 3. J Mol Biol.

[27] Pace CN, Grimsley GR, Thomson JA, Barnett BJ (1988). Conformational stability and activity of ribonuclease T1 with zero, one, and two intact disulfide bonds. J Biol Chem.

[28] McAuley A, Jacob J, Kolvenbach CG, Westland K, Lee HJ, Brych SR (2008). Contributions of a disulfide bond to the structure, stability, and dimerization of human IgG_1_ antibody CH3 domain. Protein Sci.

[29] Payne RB (1969). The controlling effect of carbohydrate in human, rabbit and bovine immunoglobulin G on proteolysis by papin. Biochem J.

[30] Youings A, Chang SC, Dwek RA, Scragg IG (1996). Site-specific glycosylation of human immunoglobulin G is altered in four rheumatoid arthritis patients. Biochem J.

[31] Krapp S, Mimura Y, Jefferis R, Huber R, Sondermann P (2003). Structural analysis of human IgG-Fc glycoforms reveals a correlation between glycosylation and structural integrity. J Mol Biol.

[32] Zheng K, Bantog C, Bayer R (2011). The impact of glycosylation on monoclonal antibody conformation and stability. MAb.

[33] Welfle K, Misselwitz R, Hausdorf G, Hohne W, Welfle H (1999). Conformation, pH-induced conformational changes, and thermal unfolding of anti-p24 (HIV-1) monoclonal antibody CB4-1 and its Fab and Fc fragments. Biochim Biophys Acta.

[34] Sathya Devi V, Coleman DR, Truntzer J (2011). Thermal unfolding curves of high concentration bovine IgG measured by FTIR spectroscopy. Protein J.

[35] Li Y, Mach H, Blue JT (2011). High throughput formulation screening for global aggregation behaviors of three monoclonal antibodies. J Pharm Sci.

[36] Martsev SP, Kravchuk ZI, Vlasov AP (1994). Large increase in thermal stability of the CH2 domain of rabbit IgG after acid treatment as evidenced by differential scanning calorimetry. Immunol Lett.

[37] Buchner J, Renner M, Lilie H, Hinz HJ, Jaenicke R, Kiefhabel T (1991). Alternatively folded states of an immunoglobulin. Biochemistry.

[38] Vlasov AP, Kravchuk ZI, Martsev SP (1996). [Non-native conformational states of immunoglobulins: thermodynamic and functional analysis of rabbit IgG]. Biokhimiia.

[39] Kats M, Richberg PC, Hughes DE (1997). pH-dependent isoform transitions of a monoclonal antibody monitored by micellar electrokinetic capillary chromatography. Anal Chem.

[40] Andrew SM, Titus JA: Purification of immunoglobulin G. Curr Protoc Cell Biol 2000, Chapter 16:Unit 16.13 (16.13.1 – 16.13.12). 10.1002/0471143030.cb1603s05 18228335

[41] Djoumerska I, Tchorbanov A, Pashov A, Vassilev T (2005). The autoreactivity of therapeutic intravenous immunoglobulin (IVIG) preparations depends on the fractionation methods used. Scand J Immunol.

[42] Djoumerska-Alexieva IK, Dimitrov JD, Voynova EN, Lacroix-Desmazes S, Kaveri SV, Vassilev TL (2010). Exposure of IgG to an acidic environment results in molecular modifications and in enhanced protective activity in sepsis. Febs J.

[43] McMahon MJ, O’Kennedy R (2000). Polyreactivity as an acquired artefact, rather than a physiologic property, of antibodies: evidence that monoreactive antibodies may gain the ability to bind to multiple antigens after exposure to low pH. J Immunol Methods.

[44] Porter RR (1959). The hydrolysis of rabbit y-globulin and antibodies with crystalline papain. Biochem J.

[45] Porter RR (1963). Chemical structure of gamma-globulin and antibodies. Br Med Bull.

[46] Edelman GM, Benacerraf B, Ovary Z, Poulik MD (1961). Structural differences among antibodies of different specificities. Proc Natl Acad Sci U S A.

[47] Edelman GM, Poulik MD (1961). Studies on structural units of the gamma-globulins. J Exp Med.

[48] Utsumi S (1969). Stepwise cleavage of rabbit immunoglobulin G by papain and isolation of four types of biologically active Fc fragments. Biochem J.

[49] Whitcomb DC, Lowe ME (2007). Human pancreatic digestive enzymes. Dig Dis Sci.

[50] Butler JE, Kennedy N (1978). The differential enzyme susceptibility of bovine immunoglobulin G1 and immunoglobulin G2 to pepsin and papain. Biochim Biophys Acta.

[51] Fang WD, Mukkur TK (1976). Physiochemical characterization of proteolytic cleavage fragments of bovine colostral immunoglobulin G1 (IgG_1_). Biochem J.

[52] Wie SI, Dorrington KJ, Froese A (1978). Characterization of the proteolytic fragments of bovine colostral IgG_1_. J Immunol.

[53] de Rham O, Isliker H (1977). Proteolysis of bovine immunoglobulins. Int Arch Allergy Appl Immunol.

[54] Reilly RM, Domingo R, Sandhu J (1997). Oral delivery of antibodies. Future pharmacokinetic trends. Clin Pharmacokinet.

[55] Petschow BW, Burnett B, Shaw AL, Weaver EM, Klein GL (2014). Serum-derived bovine immunoglobulin/protein isolate: postulated mechanism of action for management of enteropathy. Clin Exp Gastroenterol.

[56] Bosi P, Casini L, Finamore A, Cremokolini C, Merialdi G, Trevisi P (2004). Spray-dried plasma improves growth performance and reduces inflammatory status of weaned pigs challenged with enterotoxigenic Escherichia coli K88. J Anim Sci.

[57] Perez-Bosque A, Miro L, Polo J, Russell L, Campbell J, Weaver E (2010). Dietary plasma protein supplements prevent the release of mucosal proinflammatory mediators in intestinal inflammation in rats. J Nutr.

[58] Perez-Bosque A, Amat C, Polo J, Campbell JM, Crenshaw J, Russell L (2006). Spray-dried animal plasma prevents the effects of Staphylococcus aureus enterotoxin B on intestinal barrier function in weaned rats. J Nutr.

[59] Asmuth DM, Ma ZM, Albanese A, Sandler NG, Devaraj S, Knight TH (2013). Oral serum-derived bovine immunoglobulin improves duodenal immune reconstitution and absorption function in patients with HIV enteropathy. AIDS.

[60] Torrallardona D (2010). Spray dried animal plasma as an alternative to antibiotics in weanling pigs - a review -. Asian-Aust J Anim Sci.

[61] Wilson D, Evans M, Weaver E, Shaw AL, Klein GL (2013). Evaluation of serum-derived bovine immunoglobulin protein isolate in subjects with diarrhea-predominant irritable bowel syndrome. Clin Med Insights Gastroenterol.

